# The sustained “re-enchantment” of research in tropical medicine in the Brazilian Amazon

**DOI:** 10.1590/0037-8682-0406-2026

**Published:** 2026-08-21

**Authors:** Wuelton Monteiro

**Affiliations:** 1 Universidade do Estado do Amazonas, Escola Superior de Ciências da Saúde, Manaus, AM, Brasil.; 2 Fundação de Medicina Tropical Dr Heitor Vieira Dourado, Departamento de Pesquisa, Manaus, AM, Brasil.; 3 Duke University, Duke Global Health Institute, Durham, USA.

In 1917, the same year Oswaldo Cruz died, the German sociologist Max Weber delivered his famous lecture "Science as a Vocation" to the Bavarian Free Students’ Association; the expanded text was published in 1919^1^. In it, Weber defines modern science as a technical, specialized profession disconnected from tradition, subjective feeling, magic, or religion, and therefore unable to answer questions about life’s meaning. Science supplies tools for mastering technique, not for finding purpose. Within this frame the university becomes a company and the scientist an ordinary worker. Weber warned that this could lead to a "disenchantment" of the world. Scientists remain engaged by an internal, passionate commitment adapted to a bureaucratic environment that does not promise absolute truths - what Weber called a "vocation”.

It may be somewhat disenchanting to note that the historic scientific expeditions to the Amazon in 1910 and 1913, led by Oswaldo Cruz and Carlos Chagas, were planned by the First Republic’s Ministry of Agriculture, Industry and Commerce to identify barriers to economic development caused by diseases that decimated the regional workforce - with national income gains as the ultimate objective, rather than necessarily the promotion of well-being^2^. Indeed, within the period’s state bureaucracy, health actions were managed by the General Directorate of Public Health, subordinate to the Ministry of Justice and Internal Affairs^3^. Brazilian public health then followed a police-state, preventive model focused on economically important urban centers, particularly Rio de Janeiro^4^. Medical care was largely private and accessible only to those who could pay, leaving most workers without public medical coverage. This dichotomy between preventive actions and medical care persisted until Brazil’s Unified Health System was established in 1988^5^. Nevertheless, it would be anachronistic to doubt that Cruz and Chagas were compassionate toward the many malaria and leishmaniasis patients they encountered in the Amazon and the victims of yellow fever, smallpox, and plague epidemics nationwide. Actions informed by the expeditions’ data produced tangible benefits for communities - here, some measure of "enchantment" returns.

In 2015 the Revista da Sociedade Brasileira de Medicina Tropical published a supplement of 11 papers on infectious diseases in the Amazon, mainly in the state of Amazonas, many authored by researchers from the Fundação de Medicina Tropical Dr. Heitor Vieira Dourado^6^. In this new supplement we highlight the leading role this institution has played over the past decade, evidenced by many evidence-based policies: tafenoquine for radical cure of *Plasmodium vivax* malaria, decentralization of antivenom treatment for snakebites in Indigenous areas, decentralization of HIV care in Amazonas, and new drug regimens for tuberculosis and leishmaniasis, among others. The institution’s research scope has expanded to include implementation science, participatory research, Indigenous health, artificial intelligence, and omics approaches to tropical diseases. In 2026 the institution consolidated its training capacity, achieving Grade 7, the highest level of excellence in Brazil’s national postgraduate system.

Almeida et al.^7^ examine barriers to malaria control, map available resources for diagnosis and treatment, and describe actions within the Amazonas State Malaria Elimination Program. Advances in understanding the parasite-vector relationship and vivax pathophysiology are described. Studies on G6PD deficiency prevalence and the accuracy of screening tests clarify the safety profile of hypnozoiticidal drugs in the region. Pioneering implementation studies of G6PD screening and tafenoquine treatment stand out. Innovations in diagnostics and regimens are supported by pharmacogenetic data on therapeutic response and antimalarial resistance. Finally, the TELEMAL+ Program is presented as an innovative model delivering specialized care for acute febrile illness.

Sachett et al.^8^ report that scorpion stings are increasing in the Amazon and constitute a significant public health burden. This review summarizes the envenoming species, their clinical syndromes, and the temporal and geographic distribution of cases in Amazonas. Severe cases are more frequent in children and often involve circulatory collapse and acute respiratory distress. Some patients present systemic neurological manifestations, with generalized muscle spasms described as electric-shock sensations that appear refractory to available antivenom. Few studies have characterized venom composition, clinical outcomes, and antivenom effectiveness in the region.

Pinto et al.^9^ show that hepatitis B disproportionately affects populations in the Brazilian Amazon, especially Indigenous and riverine communities. Hepatitis D, associated with poor outcomes including fulminant hepatitis and rapid progression to cirrhosis or hepatocellular carcinoma, is largely restricted to this region. Although HBV vaccination has reduced incidence, the Amazon remains moderately endemic with many chronic carriers; access to adequate treatment is limited by centralization of services in Manaus and shortages of healthcare professionals in endemic areas.

Santos et al.^10^ note that despite four decades of progress in leprosy control and research in Amazonas, the disease remains a major public health challenge, with delayed diagnosis and persistent stigma. Amazonas has contributed substantially to leprosy knowledge: genomic surveillance, treatment efficacy and safety, leprosy-HIV coinfection, immunopathogenesis, and genetic susceptibility. Active transmission persists, including cases in children under 15. Ongoing challenges include limited professional training in remote areas; decentralization of primary care and telehealth integration have strengthened clinical management.

Chavarria et al.^11^ characterized viral etiologies in febrile patients in Manaus using routine testing and metagenomic sequencing. They found complex arbovirus co-circulation, dominated by dengue (DENV-1 and DENV-2) and chikungunya, with frequent coinfections. Metagenomics also detected Pegivirus hominis and Erythroparvovirus primate 1. The authors recommend integrated surveillance combining molecular, serological, and metagenomic methods.

Pinheiro et al.^12^ present an implementation analysis of tuberculosis preventive treatment (TPT) delivery in Amazonas, integrating notification data, rollout of 4R, 3HP, and 3RH regimens, adoption of IGRA diagnostics, and nurse-led decentralization. Although tuberculosis remains a major concern, these experiences illustrate how locally led science, operational innovation, and context-adapted interventions can scale up TPT and improve latent tuberculosis infection management - prerequisites for meeting national and global targets.

Barbosa-Guerra et al.^13^ review Chagas disease in Amazonas, where acute cases are mostly linked to consumption of contaminated palm fruit juices; vector-borne transmission has also been reported. Recent studies show that chronic cardiac Chagas in the Amazon has clinical characteristics and severity similar to other regions. Main challenges are logistical barriers to post-treatment follow-up and the absence of a structured network for continuous monitoring in remote areas.

Guerra et al.^14^ summarize advances in leishmaniasis control in Amazonas, noting the predominance of Leishmania guyanensis and the role of deforestation and unplanned urbanization in increasing cases. Promising clinical trial results with pentamidine, miltefosine, tamoxifen, and itraconazole are highlighted. Telehealth and treatment decentralization are proposed to improve access.

Benzaken et al.^15^ provide a comprehensive review of HIV/AIDS in Amazonas, documenting spread beyond the capital and high AIDS mortality due to late diagnosis and retention gaps, rooted in historical centralization of specialized services in Manaus. Over the last decade, expanded testing, rapid ART initiation, PrEP, HIV self-testing, and decentralized municipal services in partnership with third-party organizations have coincided with declining AIDS mortality. Tailored control measures for riverine, rural, and Indigenous contexts remain urgent.

Teixeira et al.^16^ analyze viral acute respiratory syndromes in Amazonas. Multiple respiratory pathogens co-circulate, with incidence peaks in early 2021 and 2022 during the COVID-19 pandemic and marked regional disparities. Response was highly centralized in Manaus, which coordinated transfers and laboratory diagnostics. Strengthening integrated, decentralized health systems is essential for timely responses and genomic and syndromic surveillance of emerging respiratory threats.

Monteiro et al.^17^ discuss factors behind the disproportionately high burden of snakebites in the Amazon. Riverine and Indigenous populations face the highest incidence and fatality rates yet have poorest access to antivenom. A key advance is the implementation of antivenom treatment in at least one health unit in each of Amazonas’s 62 municipalities. Pioneering culturally tailored antivenom decentralization in Indigenous health centers shows promising effectiveness and feasibility.

Santos et al.^18^ review how interdisciplinary and participatory research has advanced understanding and control of socially determined tropical diseases in the Amazon, with qualitative studies on: cultural aspects of snakebite among Indigenous peoples; acceptability and feasibility of malaria elimination interventions; stigma, gender relations, and structural inequalities affecting HIV and trachoma strategies; and perceived impacts of extreme climate events on livelihoods, disease burden, and access to health services.

Val et al.^19^ emphasize that the Amazon basin is undergoing rapid climatic and environmental change, yet we still know little about how these changes will affect tropical disease incidence. Advances in predictive modeling, remote sensing, and integrated surveillance are improving early warnings, but gaps remain in multi-stressor interactions, pathogen-vector-host adaptation, and health system resilience under climate extremes. Training qualified transdisciplinary human resources and strengthening health system adaptive capacity are crucial to anticipate and mitigate future health impacts.

Using official mortality data, Balieiro et al.^20^ found that infectious and parasitic diseases remain a major cause of death in Amazonas. A mortality peak from communicable diseases in 2019-2021 was associated with the COVID-19 pandemic. Under endemic conditions, pneumonia, HIV/AIDS, septicemia, and tuberculosis accounted for most deaths, with higher proportions among persons identified as mixed race or Indigenous. Diagnostic limitations and poor access to care contribute to disparities; high rates of nonspecific cause-of-death coding underscore the urgent need to improve death investigation.

The systematic review by Maciel et al.^21^ synthesizes metabolomics studies seeking biomarkers for diagnosis, therapeutic decisions, or pathophysiologic insight across the 20 WHO-defined neglected tropical diseases. Most studies target metabolic signatures for rapid diagnosis of leishmaniasis, dengue, schistosomiasis, and Chagas disease. Key limitations include small sample sizes and lack of methodological standardization, limiting generalizability.

Across the contributions in this supplement, we see simultaneous internationalization and local strengthening of Amazonian tropical disease research. Regional scientific production is being integrated into the global landscape while decentralizing knowledge and interventions to remote areas. Participatory, action-oriented research increasingly brings together communities, health professionals, decision-makers, and researchers to develop contextually appropriate solutions.

More than a century after Weber’s lecture, universities increasingly resemble businesses that demand productivity and efficiency. Researchers who choose to live and work in the Amazon rather than make brief field visits are unlikely to endure with a disenchanted approach to science. The logistical challenges of accessing study sites, the need to integrate traditional knowledge, engagement with the rich cultures of Indigenous and riverine communities, the exuberance of biodiversity, and the witnessing of its loss all evoke a sustained sense of astonishment - in the Aristotelian sense - and a form of "re-enchantment".
